# *Trypanosoma cruzi* down-regulates mechanosensitive proteins in cardiomyocytes

**DOI:** 10.1590/0074-02760180593

**Published:** 2019-08-15

**Authors:** Tatiana G Melo, Daniel Adesse, Maria de Nazareth Meirelles, Mirian Claudia S Pereira

**Affiliations:** 1 Fundação Oswaldo Cruz, Instituto Oswaldo Cruz, Laboratório de Ultraestrutura Celular, Rio de Janeiro, RJ, Brasil; 2 Fundação Oswaldo Cruz, Instituto Oswaldo Cruz, Laboratório de Biologia Estrutural, Rio de Janeiro, RJ, Brasil

**Keywords:** cardiomyocytes, Trypanosoma cruzi, focal adhesion, talin, paxillin, mechanotransduction

## Abstract

**BACKGROUND:**

Cardiac physiology depends on coupling and electrical and mechanical coordination through the intercalated disc. Focal adhesions offer mechanical support and signal transduction events during heart contraction-relaxation processes. Talin links integrins to the actin cytoskeleton and serves as a scaffold for the recruitment of other proteins, such as paxillin in focal adhesion formation and regulation. Chagasic cardiomyopathy is caused by infection by *Trypanosoma cruzi* and is a debilitating condition comprising extensive fibrosis, inflammation, cardiac hypertrophy and electrical alterations that culminate in heart failure.

**OBJECTIVES:**

Since mechanotransduction coordinates heart function, we evaluated the underlying mechanism implicated in the mechanical changes, focusing especially in mechanosensitive proteins and related signalling pathways during infection of cardiac cells by *T. cruzi*.

**METHODS:**

We investigated the effect of *T. cruzi* infection on the expression and distribution of talin/paxillin and associated proteins in mouse cardiomyocytes in vitro by western blotting, immunofluorescence and quantitative real-time polymerase chain reaction (qRT-PCR).

**FINDINGS:**

Talin and paxillin spatial distribution in *T. cruzi-*infected cardiomyocytes *in vitro* were altered associated with a downregulation of these proteins and mRNAs levels at 72 h post-infection (hpi). Additionally, we observed an increase in the activation of the focal adhesion kinase (FAK) concomitant with increase in β-1-integrin at 24 hpi. Finally, we detected a decrease in the activation of FAK at 72 hpi in *T. cruzi*-infected cultures.

**MAIN CONCLUSION:**

The results suggest that these changes may contribute to the mechanotransduction disturbance evidenced in chagasic cardiomyopathy.

*Trypanosoma cruzi* is the etiological agent of Chagas disease, also known as American trypanosomiasis, a disorder that affects seven million people worldwide. Even after 109 years of its discovery, Chagas disease remains neglected and a serious public health problem. Currently, this disease, found mainly in endemic areas in Latin America, spreads worldwide due to migrating populations.^1^ The parasite displays a transmission cycle that involves both an invertebrate and a vertebrate host. An infected hematophagous triatomine vector releases the infective metacyclic trypomastigote forms of *T. cruzi* in its faeces in the vertebrate host during its blood meal. The parasite rapidly invades local host cells and transforms into the replicative amastigote form. After intense replication within the host cell cytoplasm, the parasites differentiate back to trypomastigotes and promote cell lysis, reaching the bloodstream and disseminating the infection. Acute *T. cruzi* infection leads to focal myocarditis with accompanying necrosis of infected myocytes and reparative interstitial fibrosis. During the chronic disease, parasitaemia drastically decreases and parasites are barely detected. However, 30-40% of individuals can develop chronic chagasic cardiomyopathy (CCC), a debilitating condition comprising extensive fibrosis, inflammation, heart enlargement and arrhythmias that culminate in heart failure.^2^

Arrhythmogenic cardiomyopathies, such as CCC, can also be considered intercalated disc (ID) disorders, since cardiac physiology depends on the integrity of such structures for synchronised firing and contraction. Cardiac myocytes are coupled and coordinated through the ID, a junctional platform of structural and signalling molecules, such as gap and adherens junction proteins.^3^ In addition, cardiac physiology mechanobiology also involves mechanical focal adhesion properties, providing force, elasticity and signal transduction.^4^ Focal adhesions are points where extracellular matrix (ECM) components associate to the actin cytoskeleton through surface receptors, mainly integrin, and are regulated by the focal adhesion kinase (FAK) signalling pathway. FAK is a nonreceptor protein tyrosine kinase, activated mainly by integrin-dependent manner, containing a central kinase domain flanked by N- and C-terminal extensions. FAK presents an autoinhibitory FERM domain, located within the N-terminal region of FAK, which associates with the plasma membrane via its interaction with different receptors. The C-terminal region of FAK comprises the focal adhesion targeting (FAT) domain that binds directly to paxillin and talin, which in turn binds to the cytoplasmic tail of β_1_-integrins, modulating bi-directional signal transduction.^5^

Focal adhesion sites offer mechanical support and signal transduction events during heart contraction-relaxation processes. A set of mechanosensitive adapter proteins regulates cell-ECM and cell-cell adhesions during mechanical force transmission.^4^ Talin, a 50 kDa mechanosensing protein, has been shown to provide the primary link between integrin and actin cytoskeleton, as well as to participate as a scaffold for the recruitment of other proteins in focal adhesion formation.^6^ Talin structure consists of a globular N-terminal head and a large C-terminal rod that form an antiparallel homodimer. A FERM domain at the N-terminal head comprises a high-affinity binding site for the integrin β cytoplasmic domain while the rod domain (220 kDa) links to actin, vinculin and also paxillin. However, talin binding to paxillin depends on paxillin phosphorylation and their association plays an important role in focal adhesion regulation.^7^ Paxillin (68 kDa), a focal adhesion protein that was earlier observed in nascent focal adhesions at the cell periphery, is a molecular adaptor or multi-domain scaffold protein that can be phosphorylated on Tyr-31 and Tyr-118 in a FAK/Src-dependent manner. Paxillin localises to the intracellular surface of focal adhesion sites that interact with multiple signalling pathways, recruiting diverse structural and regulatory proteins. The C-terminal half of paxillin contains four double-zinc finger motifs, called the LIM domain, important in protein-protein interactions. The phosphorylation of LIM domains (LIMs 2 and 3) is required for targeting paxillin to focal adhesion. The N-terminal paxillin domain presents five leucine and aspartate rich-LD motifs that mediate proteins interactions, such as FAK and vinculin, and contains tyrosine, serine and threonine phosphorylation sites that coordinate signalling.^8^ Moreover, the N-terminal domain also comprises a proline-rich region that interacts with the vinculin-binding protein ponsin and contributes to the formation of costameres in cardiac muscle.^9^ One striking fact is that a disturbance in cardiac structural components,^10^ including costameres^11^ and junctional complexes, such as gap and adherens junctions,^12^ ^,^ ^13^ has been evidenced in *T. cruzi* infection and may contribute to the severity of the cardiomyopathy. Given the mechanosensitivity of talin and paxillin and their participation in cardiac mechanotransduction, we evaluated the expression and distribution of talin/paxillin and proteins associated during infection of cardiac cells by *T. cruzi*. Changes in this mechanosensing induced by the infection may alter the force balance across cell-ECM interaction.

## METHODS

*Cell culture and T. cruzi infection -* Cardiac muscle cells isolated from 18-day-old mouse embryos in a collagenase/trypsin solution, were plated into 24-wells for immunofluorescence or in 60 mm^2^ culture dishes for biochemical analyses. Cells were maintained in Dulbecco’s modified Eagle’s medium (DMEM) with 10% foetal bovine serum (FBS) (Cultilab, São Paulo, SP, Brazil), 2.5 mM CaCl_2_, 1 mM L-glutamine (Sigma-Aldrich, São Paulo, SP, Brazil), 2% chicken embryo extract and 1% penicillin/streptomycin solution (Life Technologies, São Paulo, SP, Brazil) at 37ºC in a 5% CO_2_ atmosphere. All procedures with animals were approved by the Comissão de Ética no Uso de Animais (Committee for the Use of Laboratory Animals) of the Instituto Oswaldo Cruz (license LW-015/17, Fundação Oswaldo Cruz). 

Trypomastigote forms of *T. cruzi*, Y strain (MHOM/BR/00/Y), were obtained from Swiss Webster mice at the parasitaemia peak, as previously described.^14^ Cardiac cells were infected at a multiplicity of 10 parasites per host cell (10:1). After 24 h of interaction, free trypomastigotes were removed and washed with Ringer’s solution (154 mM NaCl, 56 mM KCl, 225 mM CaCl_2_ pH 7.0). The infection was interrupted after 24 and 72 h post-infection (hpi).

*Indirect immunofluorescence -* Cells were fixed for 20 min at 4°C with 4% paraformaldehyde (Sigma) in phosphate-buffered saline (PBS). Primary antibodies were incubated overnight at 4ºC with anti-talin (Santa Cruz Biotechnology, Dallas, TX, USA) (1:20), anti-paxillin (Santa Cruz Biotechnology) (1:100), anti-FAK (Santa Cruz Biotechnology) (1:50) or anti-phosphorylated FAK (ThermoFisher, Bengaluru, India, 1:200), followed by incubation with secondary anti-mouse IgG-AlexaFluor555 (ThermoFisher, California, CA, USA). F-actin was visualised with AlexaFluor 488-labelled Phalloidin (ThermoFisher) and DNA was detected with To-PRO-3 Iodide (Life Technologies) or 4’,6-diamidino-2-phenylindole dihydrochloride (DAPI) (Sigma Aldrich). Slides were mounted and analysed using a confocal laser scanning microscope LSM 510 META (Zeiss) or Zeiss Axio Imager M2 microscope equipped with the Apotome system. 

*Protein extraction and immunoblotting assay -* Proteins were extracted in 50 mM Tris-HCl containing 1% Triton x-100, protease inhibitors: 10 µM E-64 (Sigma), 1 mM phenylmethylsulfonyl fluoride (Sigma-Aldrich/Merck, Darmstadt, Germany) and 1 µM pepstatin (Sigma), pH 8.0. A total of 20 μg of protein were resolved by sodium dodecyl sulfate polyacrylamide gel electrophoresis (SDS-PAGE), transferred to nitrocellulose membranes (Bio-Rad, California, CA, USA) with a transfer buffer (25 mM Tris, 192 mM glycine and 10% methanol, pH 8.3). Membranes were incubated overnight with anti-talin (1:200), anti-paxillin (1:1,000), anti-FAK (1:500), anti-pFAK (1,000) or anti-β1-integrin (1:1,000) antibodies diluted in blocking solution (5% nonfat dry milk or 1% BSA). Anti-glyceraldehyde 3-phosphate dehydrogenase (GAPDH) antibody (Life Technologies) was used as the loading control. Secondary anti-rabbit IgG or anti-mouse IgG horseradish peroxidase (HRP)-labelled antibodies (1:5,000) were incubated, revealed by chemoluminescence (Pierce/ThermoScientific) and exposed to X-ray films. Densitometric analyses were performed using the ImageJ software. The immunoblotting experiments were performed independently three times.

*Real-time polymerase chain reaction (RT-PCR) analysis -* Total RNA was extracted with Trizol (Life Technologies). One microgram of RNA was reversely transcribed into cDNA using Superscript III kit (Life Technologies). DNA contamination was excluded by prior treatment with DNase I (Qiagen, São Paulo, SP, Brazil). RT-PCR was performed using Taqman gene expression assays (Life Technologies) for talin and paxillin (Mm00659397_m1, Mm00448533_m1). GAPDH (Mm99999915_g1) was used as the normalising control. A total of 0.5 µL of cDNA was used in triplicate for each primer assay. Relative quantitative analyses were performed using the 2^-ΔΔCt^ method. 

*Statistical analyses -* Student’s *t* test was used to determine the significance of differences between mean values from at least three independent assays. A p-value ≤ 0.05 was considered significant. 

## RESULTS

**Fig. 1:**
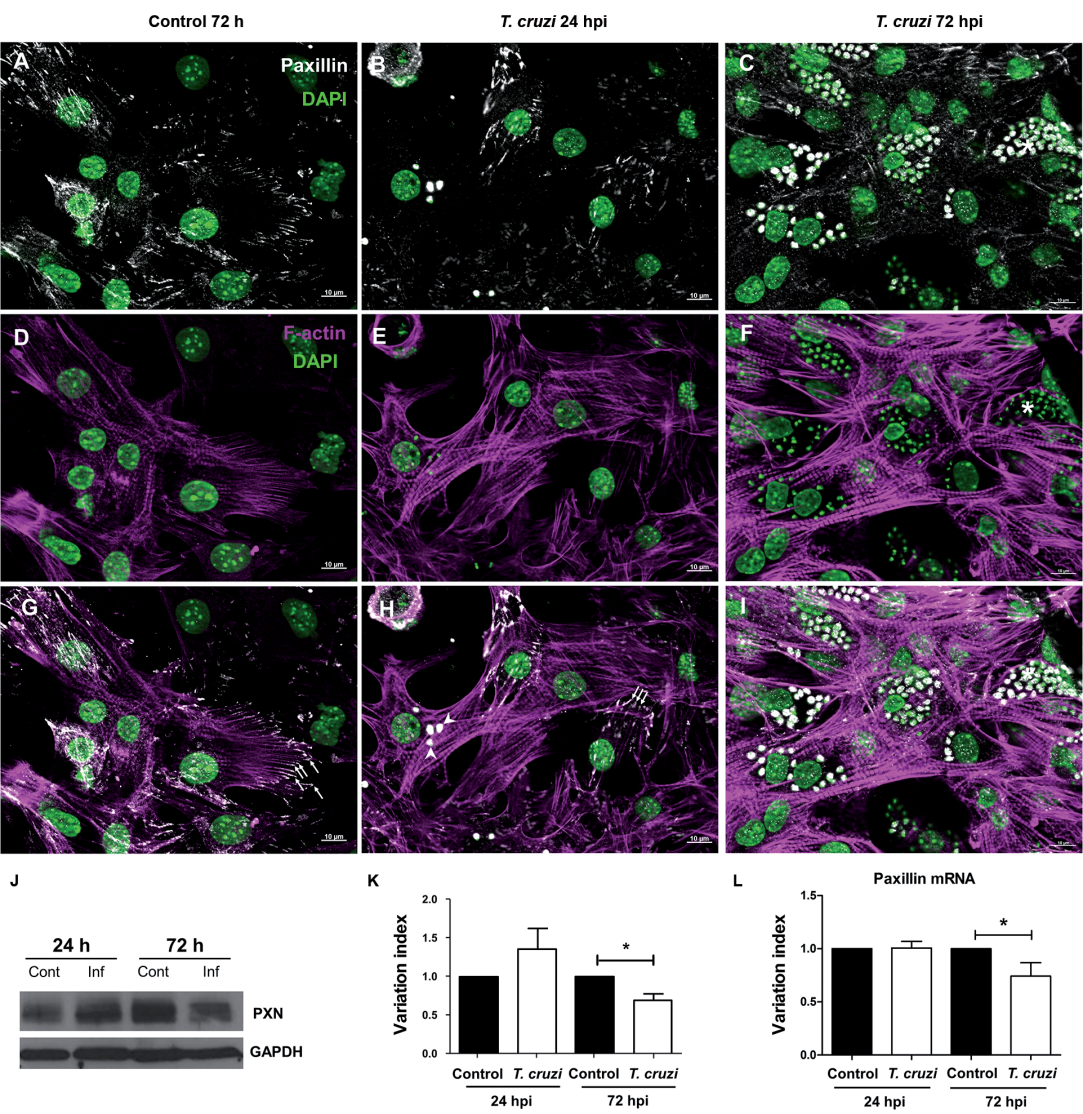
changes in paxillin induced by *Trypanosoma cruzi* infection. Double labelling of uninfected and *T. cruzi*-infected cardiomyocytes with anti-paxillin antibody (white) and To-PRO-3 iodide (green). Cardiac cells cultured for 48 h (A) and 96 h (B) displayed striations as shown by Phalloidin staining (magenta, in D-F) and paxillin staining (white in A, B and C) at focal adhesion sites (arrows in G). Paxillin location was unaltered after 24 h of infection, even in the presence of intracellular amastigotes within the host cell cytoplasm (arrowheads in B and H). Loss of paxillin in focal adhesions was observed in highly infected cells (*), concomitant with myofibrillar breakdown as evidenced by Phalloidin staining (F). Parasites were also labelled by the paxillin antibody (B and C). Paxillin content and expression were also affected at 72 h post-infection (hpi). Representative blots from three independent experiments are shown in J. Densitometric analyses revealed a 34% decrease in paxillin (K) protein content after 72 h of infection. Quantitative real-time polymerase chain reaction (RT-PCR) showed a 26% reduction in the relative expression of the paxillin transcripts (L). *: p < 0.05, unpaired Student’s *t* test. Bars: 10 µm. DAPI: 4’-6-diamidino-2-phenylindole; GAPDH: glyceraldehyde 3-phosphate dehydrogenase; PXN: paxillin.

**Fig. 2:**
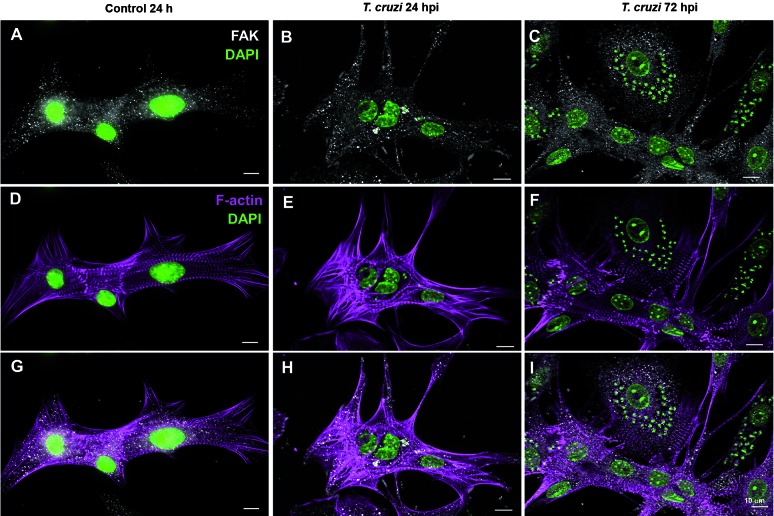
focal adhesion kinase (FAK) immunolocalisation in cardiomyocytes during *Trypanosoma cruzi* infection *in vitro*. Primary cultures were stained for total FAK protein and co-labelled with Phalloidin-Alex488 and 4’-6-diamidino-2-phenylindole (DAPI) for F-actin and nuclear visualisation, respectively. FAK immunolocalisation was found as punctate dots throughout the cytoplasm of cardiomyocytes (white in A, B and C), showing well developed myofibrils (D, E and F). Infected cells showed no significant changes in FAK immunoreactivity at 24 or 72 h post-infection (hpi) (H and I), whereas actin filaments are absent around intracellular amastigotes at later time of infection (I). Bars: 10 µm.

**Fig. 3:**
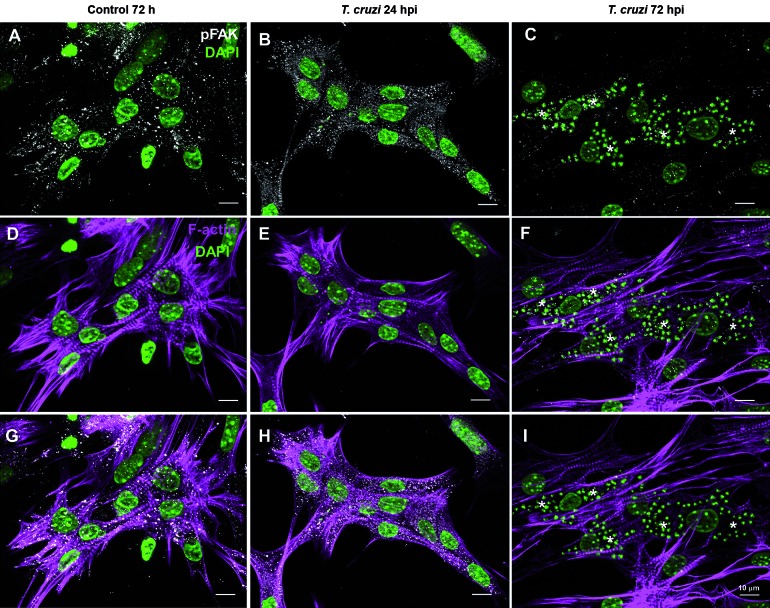
*Trypanosoma cruzi* induces a biphasic effect on focal adhesion kinase (FAK) phosphorylation in cardiac myocytes. Cultures were infected and immunostained for anti-phosphorylated FAK (white in A-C), F-actin (magenta in D-F) and 4’-6-diamidino-2-phenylindole (DAPI) for host cell and parasite chromatin (green). Intense pFAK staining was observed at 24 h post-infection (hpi) (B and H), whereas highly infected cells at 72 hpi (* in C and I) showed a clear reduction of immunoreactivity for pFAK. F-actin staining evidenced the striations of the differentiated myocytes (magenta in D, E and F). Bars: 10 μm.

**Fig. 4:**
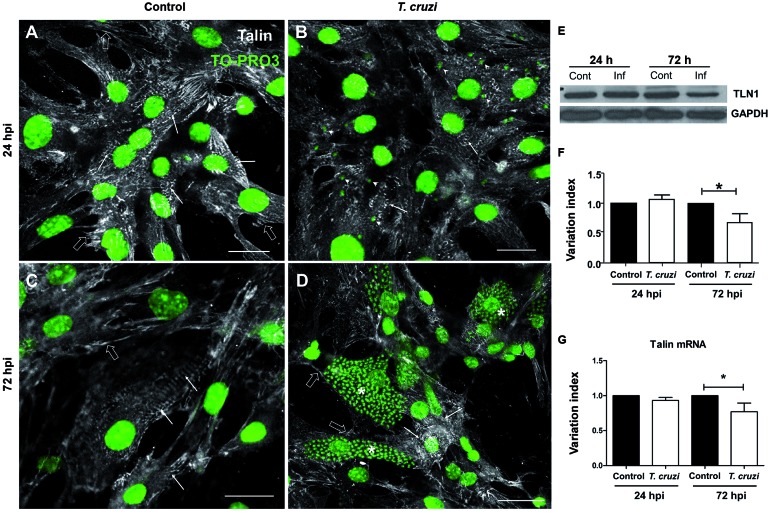
*Trypanosoma cruzi* infection disrupts talin in cardiomyocytes. Cardiac cells, cultivated for 24 h before infection with trypomastigote forms of *T. cruzi* (Y strain), were fixed at desired times and immunostained with anti-talin specific antibody (white). Nuclear chromatin was stained with To-PRO-3 Iodide (green), allowing the visualisation of host cell nucleus and intracellular parasite nuclei and kinetoplasts (arrowheads in C). Uninfected cells displayed abundant talin immunoreactivity at 48h (A) and 96h (B) *in vitro* (left panels) and revealed a striated pattern in fully differentiated myocytes (arrows), as well as staining in focal adhesion sites (opened arrows). After 24 h of infection, few intracellular parasites are seen in the host cell cytoplasm and no change is observed in talin spatial distribution (C). Talin location was drastically disturbed in highly infected cells (*), at 72 hpi, in which staining was only noticeable in focal adhesion sites. Uninfected cells in infected cultures maintained the striated talin staining pattern (D). Western blot analysis for talin (E) revealed that infected cultures had a significant decrease (32%) at 72 h post-infection (hpi) (F). Quantitative real-time polymerase chain reaction (RT-PCR) showed a 23% reduction in the relative expression of talin transcripts at 72 hpi (G). *: p < 0.05, unpaired Student’s *t* test. Bars: 20 µm. GAPDH: glyceraldehyde 3-phosphate dehydrogenase; TLN1: talin1.

**Fig. 5:**
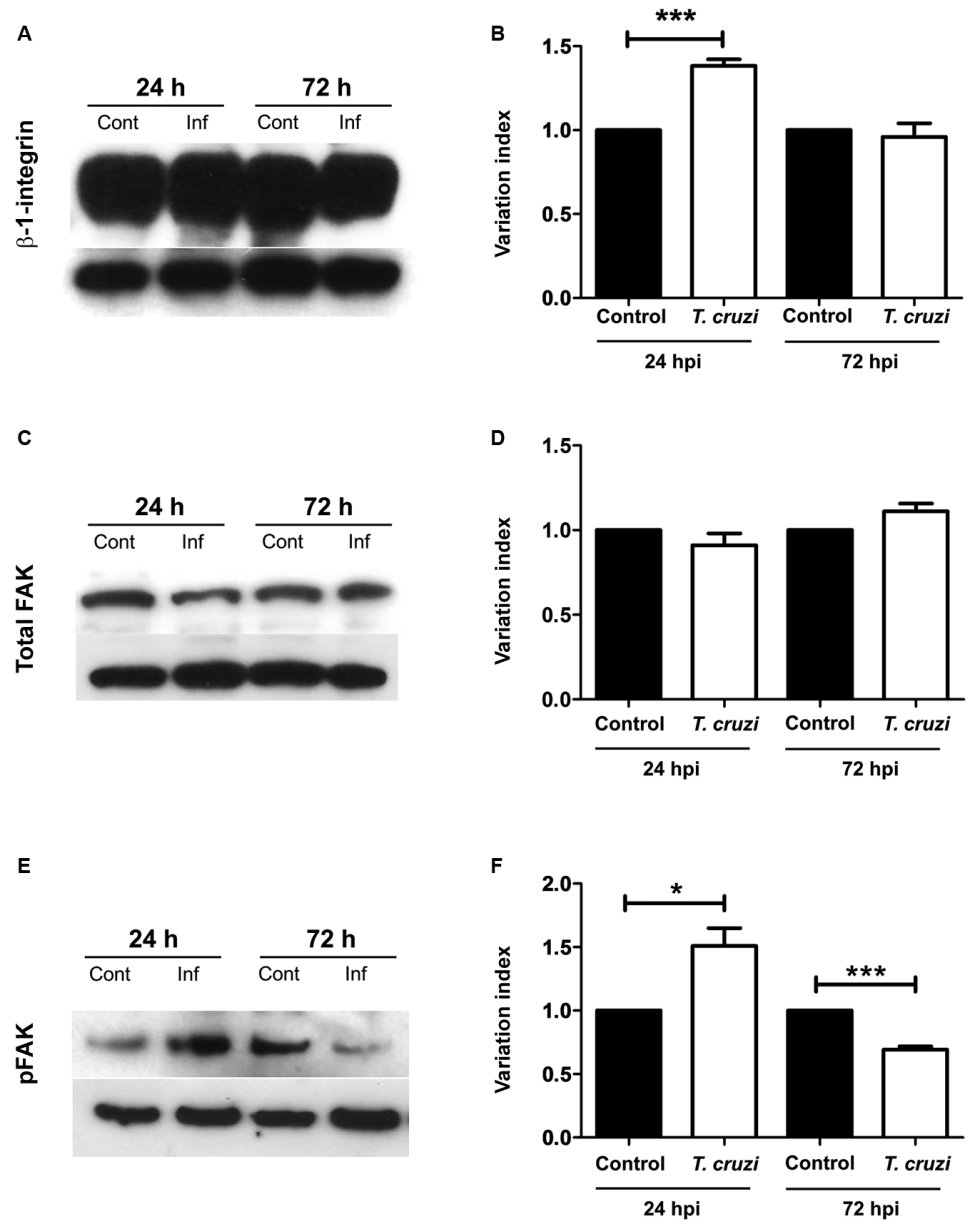
*Trypanosoma cruzi* infection alters integrin and FAK-Tyr397. β-1-integrin (A), focal adhesion kinase (FAK) (C) and FAK-Tyr397 (E) protein expression was analysed by western blot. Densitometric analyses revealed an upregulation of 38 and 50% of integrin (B) and FAK-Tyr397 (F), respectively, at the early stage of infection 24 h post-infection (hpi). A downregulation of 30% in FAK-Tyr397 expression was showed at 72 hpi (F) but no change was seen in β1-integrin (B) and total FAK (D) levels. Glyceraldehyde 3-phosphate dehydrogenase (GAPDH) was used as housekeeping control. **: p < 0.01; ***: p < 0.0001, unpaired Student’s *t* test.

The distribution of focal adhesion proteins was analysed in cardiomyocytes at the early (24 h) and late (72 h) stages of *T. cruzi* infection *in vitro*. Terminally differentiated cardiac myocytes were identified by abundant striation as shown by F-actin staining (Figs. 1-3). First, we evaluated the spatial localisation of talin and paxillin, mechanosensing and mechanosignalling proteins, respectively, in non-infected cardiomyocytes. The immunofluorescence analyses revealed both talin and paxillin located at the sites of cell-substrate adhesion (Figs. 1, 4). In addition, talin immunostaining appears as a striated pattern in cardiomyocytes (Fig. 4). Double labelling of paxillin and actin filament also revealed paxillin anchoring the ends of myofibrils in cardiomyocytes (Fig. 1). Few intracellular parasites were visualised in the host cell cytoplasm by To-PRO-3 Iodide or DAPI staining during the initial times of infection (24 hpi) (Figs. 1, 4). At this time, no alterations were noted concerning focal adhesion protein distribution. Striated talin labelling linking the myofibrils to sarcolemma and also its location at the focal adhesion sites were clearly seen at 24 hpi. Paxillin staining was also unaltered showing intense fluorescence signal at the ends of actin filaments near the edges of the cells (Fig. 1). On the other hand, with the progression of the intracellular *T. cruzi* cycle (72 hpi), both talin and paxillin demonstrated changes in their spatial distribution. Highly infected cells showed a drastic reduction of these focal adhesion proteins at attachment sites (Figs. 1, 4). Interestingly, a strong positive reactivity was observed in intracellular amastigotes by the anti-paxillin antibody. Additionally, no costameric talin staining was observed at 72 hpi (Fig. 4). These findings led us to question whether the structural changes at the focal adhesion proteins were associated with protein level alterations. Thus, we analysed talin and paxillin expressions during the course of infection by *T. cruzi*. After 24 hpi, the expression of both focal adhesion proteins remained unaffected, displaying protein levels comparable to control cells (Figs. 1, 4). The immunoblotting analysis revealed a significant decrease of both proteins at the later stage of infection (72 hpi). Reductions in protein content reached 32 and 34% in talin and paxillin expression, respectively (Figs. 1K, 4F). Next, we assessed whether the disturbances observed in protein levels correlated with transcriptional gene regulation. Quantitative RT-PCR (qRT-PCR) performed for *talin* and *paxillin* transcripts revealed that *T. cruzi* infection affects the expression of these genes, leading to a 23 and 26% reduction at 72 hpi, respectively (Figs. 1L, 4G). We also evaluated whether other proteins associated with talin and paxillin organisation, such as integrin, a transmembrane protein, and FAK, which regulates integrin-mediated mechanotransduction, undergo alterations during structural talin/paxillin disruption. Integrin and pFAK, corresponding to FAK activation, displayed 38 and 50% upregulation, respectively, at the early stage of infection (24 hpi). Interestingly, a downregulation of 30% in pFAK expression was noticed at 72 hpi, but no change was seen in β1-integrin and total FAK levels (Fig. 5). Both total and activated FAK (FAK-Tyr^397^) were also revealed by immunofluorescence. Intense FAK and pFAK staining, detected as punctate dots, was distributed throughout the uninfected cardiomyocytes cytoplasm (Figs. 2, 3). Infected cultures (24 and 72hpi) had similar immunoreactivity profile for total FAK as compared to controls. In contrast, pFAK signal was greatly reduced in highly parasitised cells at 72 hpi (Fig. 3). Myofibrillar breakdown was consistently observed in highly infected cells at 72 hpi (Figs. 1-3). 

## DISCUSSION

Mechanosignalling has been highlighted as a key feature in cardiac homeostasis. Changes in mechanosensing proteins, responsible for balancing the mechanical force between cells and their microenvironment, seem to alter mechanotransduction in pathophysiological responses.^15^ Clinical manifestations associated to biomechanical stress, such as hypertrophy, arrhythmia and heart failure, have been evidenced in cardiac diseases, including Chagas disease,^16^ which emphasises the role of physical myocardium properties in pathological process regulations. Thus, based on the fact that changes in integrin signalling, through adhesion-dependent adapter and signalling molecules, lead to abnormalities in cardiac performance, we investigated the effect of *T. cruzi* infection on mechanosensitive proteins in cardiomyocytes. 

Talin, a non-channel type protein that acts as mechanosensor, is involved in contraction force transmission by modulating cytoskeleton-integrin-ECM interaction and also triggers downstream signalling by recruiting paxillin and others mechanosensing proteins.^17^ As expected, our confocal microscopy analysis revealed talin in a striated pattern in cardiomyocytes. This finding is consistent with the presence of multiple binding sites for vinculin within the folded talin rod domain and its location in costameres as an integrin-talin-vinculin complex, allowing the transduction force from sarcomere to ECM. In fact, vinculin is essential in focal adhesion and its activation and nano-scale spatial localisation depends on the association between talin and paxillin.^18^ Additionally, paxillin location at the cell-substrate attachment site also corroborates its involvement in the regulation of focal adhesion dynamics. It has been demonstrated that paxillin phosphorylation modulates its interface with talin and triggers FAK signalling. 

At the earliest infection time (24 hpi) talin and paxillin presented no changes in their expression and distribution pattern. However, the significant increase of FAK activity concomitant with β1-integrin expression may be related to the parasite invasion process, as reported previously.^19^ The interaction of trypomastigotes, free in the culture supernatant, with integrin receptors may induce integrin clusters and trigger FAK signalling pathway to promote parasite entry. 

A remarkable finding is the lack of talin and paxillin intracellular distribution and their downregulation in cardiomyocytes induced by *T. cruzi* infection (72 h). Our data demonstrate that both mechanosensitive proteins, talin and paxillin, exhibited reduced expression levels after 72 h of infection along with massive loss of immunoreactivity in highly infected cells. Surprisingly, intracellular parasites were also reactive with the anti-paxillin antibody. Although no match for paxillin or paxillin-like proteins was identified in the *T. cruzi* genome by a BLAST analysis (http://www.dbbm.fiocruz.br/TcruziDB/), the positive cross-reaction may be related to similarities (30%) with a mucin associated surface protein (MASP). 

Several studies have pointed out the ability of many pathogens to modulate focal adhesion proteins. It has been recently demonstrated that *Leishmania amazonensis* infection disturbs macrophages migration by altering actin dynamics.^20^ Inhibition of macrophage motility seems to be driven by downregulation of paxillin and FAK phosphorylation, suggesting that the reduction of migration is responsible for the retention of *L. amazonensis*-infected macrophages in the cutaneous lesion. Although the mechanisms involved in disorganisation of focal adhesion are not completely elucidated, some are correlated with cleavage of focal adhesion proteins. The bacteria *Porphiromonas gingivales*, for example, causes paxillin proteolysis in epithelial cells.^21^ Loss of focal adhesion by ExoU enzyme activity has also been reported in HeLa cells infected by *Pseudomonas aeruginosa*, releasing talin from cell periphery.^22^ *T. cruzi* infection of cardiac myocytes leads to metalloprotease-2 and -9 activation and secretion, which results in the degradation of ECM proteins and changes in focal adhesion proteins, being correlated to the severity of chagasic cardiomyopathy.^23^ Evidence also demonstrated that collagen reduction induces paxillin and talin cleavage in smooth muscle cells.^24^ Interestingly, *in vitro* cardiomyocyte infection by *T. cruzi* greatly reduces ECM protein levels,^25^ thus suggesting a mechanism by which the infection alters focal adhesion.

We also questioned whether damage to focal adhesion proteins would be noted at the transcriptional levels. Both paxillin and talin transcripts, as well as protein levels, were downregulated, suggesting that *T. cruzi* alters mRNA regulation and, therefore, protein synthesis. Alpha-cardiac actin mRNA as well as poly(A) mRNA have also been reported to be negatively regulated by *T. cruzi* infection.^26^ Therefore, two distinct events may be proposed: (i) a deficiency in translation process due to decreased mRNA levels; or (ii) protein degradation, since it has been reported that *T. cruzi* possesses a calpain-like protein^27^ that may directly cleave both talin and paxillin. Cytokines present in the serum of infected individuals can also increase calpain activity in cardiomyocytes which, in turn, degrades structural host cell proteins.^28^ Additionally, mechanical stimulation also induces increased m-calpain expression in C2C12 cells, leading to destruction of focal adhesion proteins identified as the enzyme substrate.^29^ Considering this scenario, it is reasonable to suggest that *T. cruzi* infection leads to the proteolysis of focal adhesion components combined with transcriptional down-regulation.

Studies in the *Drosophila melanogaster* heart model, widely applied to cardiovascular system analyses, demonstrated that talin deletion results in reduced heart contractility and reported cardiac dilatation in the first instar.^30^ Talin and paxillin decrease in cardiomyocytes may be the link between the changes previously reported in *T. cruzi* infection, including cytoskeleton component,^10^ ^,^ ^31^ ECM^25^ and junctional complex^12^ disorganisation. Alteration in mechanical transduction has been previously suggested due to disturbance in costamere organisation and irregular alignment of intercalated disks in the cardiac fibres in *T. cruzi*-infected mice.^11^ Herein, downregulation of FAK phosphorylation associated to alterations in a mechanosensor protein (talin) contributes to disturbances in mechanotransduction during *T. cruzi* infection. Total FAK expression remained unaltered even at the later stage of infection (72 hpi), demonstrating a selective downregulation of FAK activity. Thus, changes in mechanosignalling proteins, namely FAK and paxillin, may result in force transmission defects and heart failure. In fact, the data from this study demonstrate that the change in mechanotransduction proteins in infected cardiac cells goes beyond the disorganisation of the spatial distribution of mechanosensitive proteins, since highly infected cultures displayed a reduction of FAK activation. Furthermore, our results suggest absence of fluorescent signal of talin at the sarcolemmal focal adhesion complexes, i.e., costameres, in highly infected cells, but remains visible at sites of focal adhesion that keep the cells adhered to the substrate. Our previous data with an experimental murine model of acute infection have demonstrated disorganisation of vinculin costameres in myocardium areas containing amastigote nests as observed in the *in vitro* model of infection.^11^ However, at the end of the acute phase, when few amastigote nests are observed, areas of intense inflammatory infiltrate in the myocardium, characteristic of the chronic Chagas cardiomyopathy, also induced changes in the costameric distribution of vinculin, suggesting that disturbances in the vinculin-talin-integrin-ECM interface may be responsible for the change in mechanotransduction in the chronic phase of Chagas disease.^11^

In summary, our data demonstrated that *T. cruzi* infection downregulates talin and paxillin expression and loss of FAK activation, resulting in integrin-mediated mechanotransduction alterations. Defects in the mechanisms of force sensing and transduction may modulate the remodelling of myocardium in response to cardiac overload. Increased knowledge of the mechanisms that activate the cardiac gene expression program may highlight new targets for drug development. Thus, further studies on mechanosensitive microRNA expression may provide new insights into the molecular mechanism underlying Chagas cardiomyopathy. 
